# The Use of an Amalgam of Mercury and Other Metals in Filling Carious Teeth

**Published:** 1870-02

**Authors:** 


### EDITORIAL DEPARTMENT.

*The Use of an Amalgam of Mercury and other Metals in Filling Carious Teeth.*—Under this heading H. M. Bowker, Esq., of Montreal, Canada, publishes the following article in the *Canada Medical Journal*:

"This compound of mercury, with other metals, has many names given to it, such as *Royal Mineral Succedaneum*, *Enamel Cement*, *Bone Paste*, *Diamond Cement*, *Lithodion*, and many others changing with the fancy and policy of the operator. Under whatever name it may appear, it has the same base article, mercury, for its principal ingredient. I know of no practice so destitute of merit, but which, at the same time, appears to have more apparent advantages than that of filling teeth with mineral paste; especially to those who are ignorant of its composition and tendencies. It is well known that mineral paste in the mouth of some patients, is productive of not only severe local disease, but the constitutional effect is such as to endanger even life. I need not refer you to the principle laid down by every chemical authority that the tendency of metals to oxydation is much increased by being alloyed. It is a question with the highest dental as well as medical authorities, whether the presence of an equal quantity of free mercury would be more pernicious than the presence of amalgam and silver, on account of the highly acid state of saliva, not only in active disease, but in some instances where there is but slight apparent deviation, from health. It is possible that there may be persons who can, with impunity, allow this "mineral paste" to remain in the mouth for a time; but there are others who cannot do so even for a few days, without leading to swelling of the glands about the tongue, throat, and neck. Neuralgia about the jaws, face and temples, even salivation and paralysis, have been produced in systems highly susceptible to the influence of mercury; for such is the difference in the idiosyncrasy of individuals, that a grain of mercury will with some, cause severe local as well as constitutional effects, whereas, with others, it requires many grains to affect them at all. I have frequently removed fillings of this "mineral paste" weighing twenty, thirty, and even forty grains, more than one half of which was mercury.

We must bear in mind that mercury and silver unite in but one proportion, and that any excess of the mercury is in a free state. When the mixture is subjected to the highest pressure in order to remove the free mercury, the amalgam then contains a preparation of sixty-four parts of mercury to thirty-six parts of silver. Such being the case, it becomes a question of great importance to know whether the oxide formed by this compound does not unite with some one of the acids or the fluids of the stomach, or of the saliva, when the system is in certain diseased conditions and form a salt of mercury which, in its mildest form, is nothing more or less than calomel. The result of the union would just as likely be corrosive sublimate as calomel.

Soon after the formation of the American Society of Dental Surgeons, which was composed of men who, for their scientific attainments and practical skill, were unsurpassed, a resolution was unanimously carried to the effect that "this Society regard the use of 'mineral paste' for plugging carious teeth as malpractice." A similar resolution was passed by the Medical Society of New York.

I know of many patients who have been treated by their physicians for certain diseases caused by amalgam plugs in the mouth, when neither the physician nor the patient suspected the cause. Many cases of what are called "Spontaneous Salivation," have been produced, and are the legitimate result of the presence of amalgam plugs in the teeth, for as soon as the mouth is relieved of the pernicious compound, all the unfavorable symptoms pass away, therefore, no further doubt can exist respecting the cause of the malady.

The question is often and naturally asked why this amalgam is so generally used by a certain class of dentists.

The answer can be found in one or all of the following explanations:

1st. The cheapness of the material.

2nd. The ease and facility with which it is used; for it can be put into the most difficult cavities with as much ease as so much putty or wax.

3rd. It makes up for the want of skill and ability to use something better.

4th. From ignorance or the want of honesty.

Were it not for these objections and others which might be given, I would unhesitatingly make use of the "paste" in my own practice, for by so doing, I should save labor, time, money, and derive as much profit and advantage as those who persist in using it. But if it can be proved that this mineral paste is constitutionally injurious, in any single case, its use should be abandoned by all who take an interest in the standing of their profession, or have any regard for their reputation

It is with much reluctance that I appear as the expositor of the abuses of dentistry: But were I to remain silent, I should consider it would be a violation both of duty and conscience, and when I see an institution, such as exists in Toronto, with the imposing title of the "Royal College of Dental Surgeons," encouraging the use of such a pernicious compound, and that the same may be said of the "Dental Association of Quebec," I think it time that the public should clearly understand the risk the patient runs by the use of it. As I have remarked, the highest dental and medical authorities, both European and American, have condemned the use of amalgam, in any form whatever, for filling teeth, as malpractice. Yet in spite of this positive dictum the Dental Societies of Canada, who put themselves forward as the guardians and representatives of the profession in the Dominion, not only advocate but vindicate its use.

The question in its effects becomes medical, and clearly within the sphere of the journal under your direction; therefore I respectfully ask the assistance of the leading members of the faculty to discountenance what has been proved to be a most pernicious practice."

This article is somewhat severe upon the "Royal College of Dental Surgeons" and the Dental Societies of Canada. Who Mr. Bowker is we do not know, perhaps the *Canada Dental Journal* can enlighten us; but whether Mr. B. is qualified by professional experience and investigation to make a report upon this subject or not, much that he says is true, but at the same time we think that he has taken an extreme view of the case.

Although no advocate for the indiscriminate use of amalgam, and believing that tin-foil is much superior as a cheap material for filling teeth, yet we think this compound may be used in teeth which are mere shells, so far gone that no other metal can be safely introduced, and that it will preserve such teeth for a time at least, especially where their extraction is contra-indicated for some good reason.

On the other hand such fillings should never be used in teeth which it is possible to fill with either gold or tin-foil ; and in no case should amalgam be used in front teeth, or in the pulp cavities of teeth, or in the proximity of a living pulp.

When properly prepared and properly introduced, instead of amalgam fillings containing sixty-four parts of mercury to thirty-six parts of silver, as Mr. B. asserts, the proportion of mercury need not, and should not be half so great.

The objections urged against this compound in Mr. B.'s article would certainly hold good, if the amalgam used at the present time was as impure as that employed ten or twelve years ago. But a great improvement has been made, not alone as regards the purity of the ingredients composing amalgam, but also as to the manner of preparing and introducing it into the teeth.

The following is the best method for using this material in cases where its use is indicated :

" Mix and incorporate the Fillings with *Redistilled Mercury,* in the usual way, to the consistency of a stiff paste. Wash thoroughly with Alcohol, adding a few drops of a strong solution of Chloride of Zinc, and remove any excess of Mercury by twisting in a piece of chamois skin or stout muslin, then with a pair of large Flat-nose Plyers, press the mass until about the thickness of an ordinary knife-blade, and cut into squares or blocks suited to the size of the cavity to be filled. Should the Amalgam be too dry to work satisfactorily, warm the instrument used slightly over a spirit-flame, and apply steadily to the mass in the cavity for a few moments, and the desired object will be attained."

" The squares or blocks can be best conveyed to the cavity with a rough surface instrument, but should be worked with a smooth surface plugger, slightly heated by passing through a spirit-flame, or dropping in hot water."

" It is a great mistake to suppose, as many do, that it makes but little difference what kind of an instrument is used in plugging with Amalgam. It is just as necessary to have instruments especially adapted to this work as it is for any other description of filling Next to the use of Amalgam of inferior quality, there is doubtless nothing that has caused so much dissatisfaction in its use as the want of suitable instruments."

" The same care should be given to the preparation of a cavity for an Amalgam filling as for Gold. The edges should be nicely beveled, the cavity thoroughly dried, and slightly touched with Creasote. In the event of a near approach to the pulp, it is best to guard against after trouble by placing a small quantity of Asbestos enveloped in Tin-foil to cover the base of the cavity."

"It is important to keep the cavity dry, if possible, during the operation. The Amalgam should be condensed firmly from base to surface."

"When the filling is sufficiently hard, finish and burnish as carefully as the best quality of Gold filling."

